# Exploring individual's public trust in the NHS Test and Trace System – A pragmatic reflexive thematic analysis

**DOI:** 10.1016/j.invent.2024.100740

**Published:** 2024-04-04

**Authors:** C.M. Babbage, H. Wagner, L. Dowthwaite, V. Portillo, E. Perez, J. Fischer

**Affiliations:** a NIHR HealthTech Research Centre in Mental Heath (MindTech), School of Medicine, University of Nottingham, Nottingham, United Kingdom; b School of Computing, Engineering & the Built Environment, Edinburgh Napier University, Edinburgh, United Kingdom; c Horizon Digital Economy Research, University of Nottingham, Nottingham, United Kingdom; d Mixed Reality Lab, School of Computer Science, University of Nottingham, Nottingham, United Kingdom

**Keywords:** COVID-19, Digital contact tracing, Public trust, Reflexive thematic analysis, Responsible research and innovation, Patient and public involvement

## Abstract

**Context:**

Digital contact tracing uses automated systems and location technology embedded on smartphone software for efficient identification of individuals exposed to COVID-19. Such systems are only effective with high compliance, yet compliance is mediated by public trust in the system. This work explored the perception of individual's trust and expectation of the broader Test and Trace system in the United Kingdom (UK) with the upcoming release of the National Health Service's (NHS) COVID-19 app as a case example.

**Methods:**

Twelve adults underwent online semi-structured interviews in August 2020, prior to public availability of the COVID-19 app. Pragmatic reflexive thematic analysis was applied inductively to explore common themes between participants, using an organic and recursive process (Braun & Clarke, 2019).

**Results:**

Themes highlighted features of the technology that would be perceived to be trustworthy (Theme 1), and concerns relating to i) whether users would comply with a T&T system (Theme 2) and ii) how a T&T system would handle user's personal data (Theme 3). Two further themes built on aspects of automation within a T&T system and its impact on trust (Theme 4) and how the media altered perceptions of the T&T system (Theme 5).

**Conclusions:**

Participants outlined the need for different user requirements that could be built into the NHS COVID-19 app that would support increased adherence. Concurrently, participants raised questions surrounding personal data and privacy of their data, plus the level of automated versus manual tasks, which impacted perception of trust in the app and wider system. Additionally, themes highlighted that T&T systems do not happen within a vacuum, but within a pre-existing environment influenced by variables such as the media and perception of other's compliance to T&T.

**Implications:**

Since it's roll-out, controversies surrounding the UK T&T system include concerns about privacy, stigma and uptake. Considering the current piece of work, which anticipated similar concerns prior to public access to COVID-19 app, engaging with the public may have been an important step in improving the perception and compliance with the app. Principles fundamental to patient and public involvement (PPI) and Responsible Research and Innovation (RRI) such as the inclusion of the public in the early development of research and aligning the outcomes of research and innovation with broader societal values and expectations would have been well-applied to this system and should be applied to future autonomous systems requiring high public uptake.

## 1 Introduction

The World Health Organisation declared the COVID-19 outbreak a pandemic in March 2020. Prior to medical intervention availability (e.g. vaccinations), contact tracing was recommended as a crucial technique for controlling the spread (Kendall et al., 2020). Four months later, in the United Kingdom (UK), the National Health Service (NHS) Test and Trace programme (T&T) was launched leading to a pilot of the COVID-19 app digitising contact tracing being tested on the Isle of Wight, a small island off England's South Coast (Kendall et al., 2020). In September, the NHS COVID-19 app (hereon called ‘COVID-19 app’) became publicly available (Department of Health and Social Care, 2020).

Contact tracing uses identification and quarantining of individuals exposed to a disease to interrupt the spread of the disease (World Health Organization, 2021). During the severe acute respiratory syndrome (SARS) epidemic in 2003, well-implemented contact tracing was credited with containing its spread (Kendall et al., 2020). This traditional approach relied on officials questioning individuals about who they had been ‘in contact’ with and requested them to isolate if they had come into contact with anyone with SARS (Kendall et al., 2020; Bell et al., 2004). A digital approach uses a smartphone app to record location e.g., through GPS (global positioning systems), Bluetooth or QR (quick-response) codes and symptom recording, to identify if an individual has come into contact with COVID-19 (Anglemyer et al., 2020). Automated digital contact tracing may be advantageous over manual and memory-reliant methods, considering the highly efficient rate of transmission of COVID-19 (Kendall et al., 2020).

Previous pandemics have highlighted the importance of trust in healthcare systems for increasing public compliance, risk management and therefore, effectiveness (Siegrist et al., 2021; Siegrist and Zingg, 2014). Yet the literature around public trust is vast; definitions within the topic are contested and empirical study of trust remains challenging (Taylor et al., 2023). The definition of ‘public trust’ has reached some consensus as a social phenomenon, basing it on shared norms, individual (or interpersonal) trust, developed through both communication and activities e.g., citizen juries or through developing social capital (Gille et al., 2017). An understanding of ‘public trust in healthcare systems’ requires application of public trust to a health care context. Current conceptualisations, though still debated, recognise the influence of personal experience with healthcare or healthcare professionals, and wider variables such as outside organisations, the government or ‘state’, or public spheres of influence (Gille et al., 2017; van der Schee et al., 2007). Models also account for how perception is shaped, e.g. through media, or communications and also how these domains can influence one another Together, these definitions and conceptualisations highlight the complexity within the literature which can make application of trust to real-world settings difficult.

The COVID-19 pandemic, with reports of vaccine hesitancy and reduced uptake of the COVID-19 app, has amplified the need for greater understanding of trust-building within healthcare systems (Siegrist et al., 2021; Siegrist and Zingg, 2014; Taylor et al., 2023). An exploratory analysis of correlates for COVID-19 prevention and treatment across 177 countries indicated that higher levels of interpersonal trust and trust in the government had large significant associations with lower levels of infection. Other than governmental corruption, no other social factors were identified as having an association with infection rates (Bollyky et al., 2022). Furthermore, trust has been found to have a significant effect on health outcomes (Kelley et al., 2014), for example compliance, a trust-sensitive variable, has been found to be three times higher when primary care relationships are characterised by high levels of trust (Martin et al., 2005; Birkhauer et al., 2017). During a pandemic, risk and uncertainty are heightened, furthering the need for trust in healthcare (Calnan and Rowe, 2006). Consideration of trust across sectors of society also deserves when certain groups might display differing levels of trust in e.g., technology, such as the elderly (Weck and Afanassieva, 2023) or in government and healthcare, such as black and minority ethnic groups (Taylor et al., 2023). This is especially important given both these groups were considered to be at greater risk of harm from COVID-19 (Calnan and Rowe, 2006; Perrotta et al., 2020; Aldridge et al., 2020).

Furthermore, whilst data collection is commonplace in healthcare settings for recording medical and personal information (Damschroder et al., 2007), the national emergency acted as a stimulus to tracking and data-collection previously never seen by health care providers. Tracing through smartphones relies on individuals giving up personal information not usually supplied, or supplied as frequently to healthcare providers, including: covid-status, exposure to covid, social contacts, location and covid-status of social group (Redmiles, 2021). As privacy violations or misuse of personal data are one of the main concerns related to trust in technologies (Toreini et al., 2020), it is not surprising similar concerns were also highlighted with the COVID-19 app (Williams et al., 2020). Considering the necessity of compliance to the effectiveness of the COVID-19 app (Ferretti et al., 2020), understanding what can be done to improve trust is essential to controlling the spread of COVID.

Trust is also implicated when new technologies are presented to society, as understanding the general public's views of technology and research innovation is important to improve the perception and uptake of new ideas. Responsible Research and Innovation (RRI) aims to align the processes and outcomes of research and innovation with broader societal values and expectations (Nielsen et al., 2018; Pacifico Silva et al., 2018). RRI is an attempt to develop a framework to support societal readiness for new technologies that are ethically acceptable, sustainable and socially desirable (Pacifico Silva et al., 2018). Therefore, the inclusion of the public, stakeholders and societal actors, alongside funders and researchers is essential during innovation (Nielsen et al., 2018). Outside of RRI, various guidelines exist to promote the inclusion of patient and public involvement (PPI) in the early development of funding applications and research (NIHR, 2019; Craig et al., 2008; Yardley et al., 2015; Mohr et al., 2013). Iterative approaches used in PPI improve engagement, acceptability, and feasibility of interventions, as they highlight weaknesses and refinements prior to the implementation of and improve efficiency when delivering interventions in different settings and to populations (Thabrew et al., 2018; Bevan Jones et al., 2020; Garrido et al., 2019; Grist et al., 2019; Hollis et al., 2016). Together, RRI and PPI share similar principles that support increased uptake and support of new technology innovations. Given concerns raised in an open letter signed by over 170 academics with expertise in privacy and security (Percy, 2024; Digital Health, 2024) and public concerns highlighted around stigma and uptake of a hypothetical contact-tracing app (Williams et al., 2020), engaging with the public would have been an important step in improving the public perception and compliance with such a system.

The aim of this piece of work was to explore individual's perceptions of trust in automated systems, with the COVID-19 app acting as an example of such a system. Interviews were used as a method to gather in-depth understanding of individual's opinions, particularly in relation to concerns and reassurances regarding an app. Initially, this work was carried out to support the formation of a large-scale questionnaire for quantitative data collection once the app was available (see (Dowthwaite et al., 2021a)). Nonetheless, these interviews contained rich data and findings that were felt to provide a unique insight into individual's expectations and perceptions of the NHS COVID-19 app ahead of its availability and the data.

## 2 Material and methods

### 2.1 Participants and recruitment

Twelve adults were recruited opportunistically via email and social media to take part in online video-recorded interviews conducted by LD, a female Research Fellow with 11 years' experience in qualitative research. All who responded to the call were interviewed. A pilot interview was completed prior to the interviews to ensure the interview guide and process flowed well. Participants were located throughout the UK, in a private setting during the interview: 2 in Wales, 2 in Scotland, 8 in England. Participants were offered a £15 Amazon voucher for taking part in the research.

The research was approved by and adhered to the University of Nottingham, School of Computer Science Research Ethics Committee (ref: CS-2019-R57).

### 2.2 Study procedure

After receiving an information sheet, interested participants contacted the researchers to express their interest in being interviewed. Participants completed online consent forms and were contacted to organise a time for their interview. Interviews were hosted and video recorded via Microsoft Teams. Interviews were initiated by gaining verbal consent to take part in a recorded interview and an overview of the process. During the interview, an interview guide was followed in a semi-structured manner and interviews lasted between 25 min and 1 h 12 min (mean = 00:46:20). The consolidated criteria for reporting qualitative research (COREQ) (see Appendix 3) and the standards for reporting qualitative research (SRQR) have been followed to ensure transparent and quality reporting (Tong et al., 2007; Reed et al., 2014).

### 2.3 Development of interview guide

The semi-structured interview guide can be seen in Appendix 1. The interview guide started with broad questions about the current system T&T system, the ecosystem of the pandemic, other countries, the NHS and the government before moving onto questions around technology to understand public perceptions of trust in autonomous systems.

## 3 Analysis

Thematic analysis sits within a qualitative epistemology and includes an organic and iterative process which enables the researcher to return to earlier steps before finalising their analysis. This ensures the themes feel stable at the end of the analysis period. Thematic analysis requires reflection and active engagement with the data, using codes as a means to explore the rich detail held within the data (Swain, 2013). A pragmatic method was used taking influence from theme-based content analysis and codebook approaches with the use of codebooks, member checks and systematic documenting of the process (Braun and Clarke, 2006). Such methods are not readily associated with reflexive processes due to their positivist associations nevertheless, this approach supported understanding and transparency for a multi-disciplinary research team with multiple coders in the project, completing analysis during a pandemic ‘working from home’.

Analysis was completed by CMB and HW who have experience of conducting and writing qualitative research. Analysis was completed taking an inductive approach, broadly following a recursive process with the thematic analysis steps (Braun and Clarke, 2013) using spreadsheet software. In brief, these steps include formatting the transcripts into spreadsheets and applying initial codes, concise summaries of a topic, to each line of conversation relating to the aims of the research throughout the transcript. Once initial codes are formed, they can be grouped into initial themes based on both frequency and depth of codes, continuing until broader themes and subthemes become refined and defined. Spreadsheets allowed the researchers to collaborate together, whilst holding mass amounts of data, with the ability to organise and ‘cut and paste’ the data (or codes and initial themes) with flexibility, and to save and store the process. Member checks, a process to identify and resolve confounding codes or themes, were completed at each stage by CB and HW, with regular involvement from other team members for sense checking, particularly with LD who carried out the interviews. Categorisation of themes was explored but were not felt to bring any further stabilisation for the themes, and so were not used.

### 3.1 Reflective statement

Reflexivity is an important part of qualitative research; data is not coded in epistemological vacuum and individual researchers have their own, differing perspectives and experiences (Braun and Clarke, 2019; Braun and Clarke, 2013). To maintain a reflexive approach, CB and HW kept field notes and discussed potential influences their thoughts and processes may have during the analysis. LD and VP also shared notes they gathered during data collection and transcription.

## 4 Results

Interviews were completed with 12 adult participants; 6 female, 6 male. Ethnicity was given by 11 participants; 9 white British, 1 Indian and 1 Pakistani. For religion, 8 participants recorded no religion, 2 were Christian, 1 was Hindu and 1 was Muslim. The highest qualification for the participants included 5 undergraduate degrees, 4 master's degrees, 1 PhD and 2 ‘other’. Breadth, i.e., new potential themes, depth i.e., deep analysis of potential themes of the data and pragmatism i.e., resources required given the need to move onto the next stage of research, were considered by the research team when deciding on the number of participants to interview. Following recommendations for thematic analysis, a pool of 12 participants is viewed as acceptable for medium sized studies (Braun and Clarke, 2019) and given the specific topic area, the data was felt to be rich enough to be suitable for thematic analysis.

### 4.1 Overview of themes

**Fig. 1:**
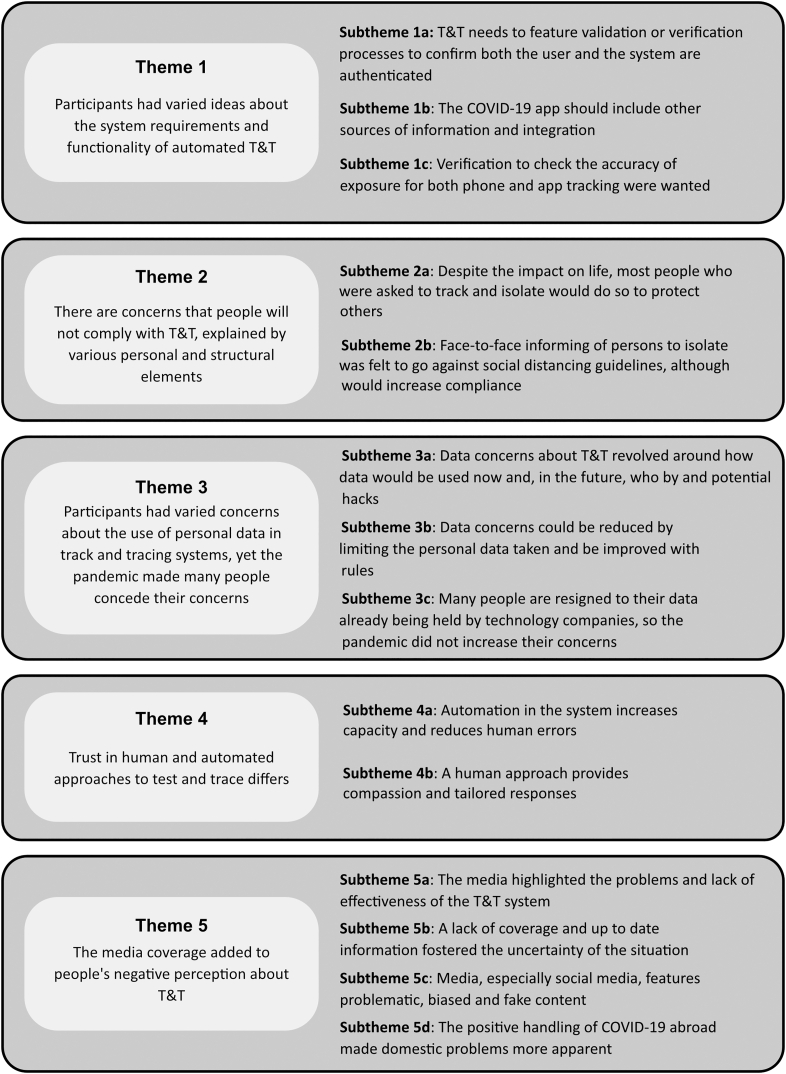
An overview of the five themes presented with their subthemes.

Five themes are presented that were developed from the data, for an overview see Fig. 1. Supporting quotes for each of the themes and subthemes can be viewed in Appendix 2. Themes highlighted how participants thought T&T technology could be developed to be trustworthy and useful (Theme 1) and questioned whether they and others would comply with a T&T system (Theme 2). Concerns regarding the handling of personal data in tracing systems constituted a further theme (Theme 3). Finally, Theme 4 related to aspects of automation within a T&T system and its impact on trust, and the perception of T&T delivered by the media was provided in Theme 5.

### 4.2 Theme 1: participants had varied ideas about the system requirements and functionality of automated T&T

Participants expected T&T would help identify potential transmissions of COVID-19 but there were varied ideas regarding how T&T should work. Subthemes of Theme 1 therefore included the need for some form of validation or verification process of the tracing system (1a), integration of additional information in the COVID-19 app in addition to contact tracing (1b) and methods to check the accuracy of the exposure (1c).

#### 4.2.1 Subtheme 1a: the COVID-19 app needs to feature validation or verification processes to confirm both the user and the system are authenticated

The need for validation of a genuine product begins as early as downloading the application; the user needs to feel able to trust the developers, wants to see that the COVID-19 app is authenticated and needs to know who will be contacting them. If persons were to be contacted by a phone call, extra verification processes were felt to be necessary.

#### 4.2.2 Subtheme 1b: the COVID-19 app should include other sources of information and integration

Participants highlighted how additional features would make an app-based solution more attractive. For example, including fitness trackers such as a step-count or the addition of further information such as guidance for what to do if the user receives a T&T notification were suggested. Participants were concerned about the COVID-19 app not including further advice for if they had become infected. Finally, participants wanted features that could create a more individualised app environment such as being able to share information with your bubble and accessing information about the local situation.

#### 4.2.3 Subtheme 1c: verification to check the accuracy of exposure for both phone and app tracking were wanted

In addition to a verified T&T system or technologies (Subtheme 1a), participants also wanted ways to check the accuracy of a request to isolate. Details of one's exposure to Covid-19 was desired including location, distance from exposure origin and length of exposure. Participants intended to use this information to verify the system alters, where inaccurate information or false positives could lead to mistrust.

### 4.3 Theme 2: there are concerns that people will not comply with T&T, explained by various personal and structural elements

In theme 2, participants were concerned about non-compliance with T&T. Potential reasons that were given for non-compliance included personal and structural elements. At the personal level, factors included a lack of access to technology, effort to engage, pandemic fatigue and personality. Codes were congregated around a lack of regulation and the T&T system being easy to ignore, as structural reasons about potential public non-compliance. Nonetheless, as highlighted by the subthemes, participants within the study resonated that they would be happy to isolate to protect others (2a) and expected the system would not use face to face communication, to support social distancing guidelines (2a).

#### 4.3.1 Subtheme 2a: despite the impact on life, most people who were asked to track and isolate would do so to protect others

When queried on whether they would comply with a T&T system, a strong sense of concern for others was noted, particularly regarding support for the benefit of the public, rather than oneself. Participants talked about concerns they had in spreading the virus unaware, and therefore a concern for others pre-empted compliance with T&T.

#### 4.3.2 Subtheme 2b: face-to-face informing of persons to isolate was felt to go against social distancing guidelines, although would increase compliance

A door-knocking or face-to-face notification of exposure to Covid-19, was not considered to be appropriate by participants due to the potential for contamination.

### 4.4 Theme 3: participants had varied concerns about the use of personal data in track and tracing systems, yet the pandemic made many people concede their concerns

Three subthemes were developed relating to Theme 3, which centred on concerns that people had in using T&T systems where their personal data was involved and highlighted how this varied amongst participants. Concerns were dependent on how personal data was being used and who by (3a) and participants made suggestions for what could be implemented to reduce these concerns (3b), yet for participants who already felt their data was being held elsewhere were acquiescent about their data-use (3c).

#### 4.4.1 Subtheme 3a: data concerns about T&T revolved around how data would be used now and, in the future, who by and potential hacks

Many participants reported that the way their personal data was being used was important in whether they had concerns about its use. A requirement often stated by participants included the use of data for the specified purpose and duration only.

#### 4.4.2 Subtheme 3b: data concerns could be reduced by limiting the personal data taken and be improved with rules

Reducing the unnecessary personal data retrieved and having rules in place was felt to lead to fewer concerns with T&T systems. Participants did not believe it would be necessary for T&T to know the exact location of their location or what they had been doing. Similarly, the details required to log in to the COVID-19 app should correspond to the purpose of the app and it was queried if other details should be taken. Finally, participants wanted tech companies to abide by governmental laws and be held accountable by independent regulators.

#### 4.4.3 Subtheme 3c: many people are resigned to their data already being held by technology companies so the pandemic did not increase their concerns

Some participants already felt their data was being held by technology companies and had resigned to thinking there had already been a breach of their data. These participants also appeared content with others holding their personal data and in light of T&T did not display any increased concern about data-use.

### 4.5 Theme 4: trust in human and automated approaches to test and trace differs

Whilst trust was identified as integral to the T&T system, in Theme 4 participants are shown to have varied opinions on whether they would prefer a system that communicates with them automatically or via a human approach. Participants overwhelmingly identified the former as improving the capacity and accuracy of the system (4a) however some felt the latter conveyed more compassion than automated approaches (4b).

#### 4.5.1 Subtheme 4a: automation in the system increases capacity and reduces human errors

Most participants felt a computer system such as an app would be more trustworthy than an alternative approach, with reasons for greater trust in this system including automation having greater capacity, such as being able to contact people more quickly and being more reliable. Automation also overcomes variability in the system and makes it more feasible for persons to easily comply.

#### 4.5.2 Subtheme 4b: a human approach provides compassion and tailored responses

On the other hand, automated systems were sometimes not trusted by participants owing to the lack of human approach. A human approach was felt to provide compassion that was felt necessary for the content of the messages being transmitted by T&T that could potentially be bad news. This held true, despite an awareness of the scale needed for a T&T system. Furthermore, tailored information could be given in a human-approach if needed.

### 4.6 Theme 5: the media coverage added to people's negative perception about T&T

Theme 5 highlights the role of the media in how opinions were developed and formed related to T&T, with a negative perception being compounded by comparisons with different countries, who appeared to be handling covid better than the UK. The media was held especially responsible for bringing greater awareness of the problems surrounding T&T (5a), a lack of up-to-date information (5b) and social media as a heightened source of unreliable information (5c).

#### 4.6.1 Subtheme 5a: the media highlighted the problems and lack of effectiveness of the T&T system

Reading about T&T in the media was often associated with learning about its shortcomings such as concerns about how effective the T&T system would be. As a result, participants didn't feel the system was of the same quality to other systems, felt people would not comply with the system, and raised questions about how the intervention would be staffed.

#### 4.6.2 Subtheme 5b: a lack of coverage and up to date information fostered the uncertainty of the situation

A lack of coverage or exposure about the T&T system in turn led to greater distrust in the system, with several participants commenting on the overall lack of coverage or up-to-date information as the pandemic progressed. Even regularly consuming news sources that were viewed as more superior, was of no help, leading participants to feel unable to predict the future. Participants called for more information about positive behaviours or the use of advertisement campaigns to increase trust in the T&T system.

#### 4.6.3 Subtheme 5c: media, especially social media, features problematic, biased and fake content

Social media was most often grouped into featuring potentially problematic, false, or biased information. Participants were critical about the way numbers could be misrepresented through reporting with a lack of context or point for comparison. Additionally, participants recognised sources were often biased in one way or another. However, the abundance of information and people not fact-checking on social media, was noted to be especially difficult to deal with. Furthermore, social media was noted to be a place of extremes where fake news or conspiracies were numerous.

#### 4.6.4 Subtheme 5d: the positive handling of COVID-19 abroad made domestic problems more apparent

Participants would often compare with other countries, often perceiving the British situation to be worse. Participants spoke of countries having more effective T&T systems that they thought had been implemented more successfully, exposing the problems in the UK. Cultural differences between societies were given as a reason for why other societies had purported higher adherence to COVID-19 prevention measures. Politics and cultural norms were also recognised for their role in how a country handles a pandemic.

## 5 Discussion

The outbreak of COVID-19 led to the development of digital contract tracing systems to identify individuals exposed to COVID-19 as a method to control the spread of the disease. A digital approach to contact tracing offers a more efficient method over manual methods (Kendall et al., 2020), yet data collection at the mass scale required for COVID-19 was a first for health care providers. Trust of healthcare providers and governments is a known mediating factor in compliance, especially relating to medical conditions (Lee and Lin, 2008). Prior to the deployment of the COVID-19 app, this research group carried out interviews with the public to understand their perceptions of T&T, particularly in relation to trust. A pragmatic reflexive thematic analysis of the data led to the generation of five themes. Specifically, participants raised concerns about how well digital contact tracing could work for the purpose of tracing a virus (Theme 1) and questioned if the public would adhere to such systems at all (Theme 2). Participants queried the functionality of the COVID-19 app (Theme 4) and concerns regarding the use of participant's personal data in a T&T system were put forward in Theme 3. The final theme (Anglemyer et al., 2020) showed that media portrayed a negative perception of the T&T system and handling of the pandemic. These themes highlighted that the implementation of a contact tracing system for an infectious disease does not happen within a vacuum, but there is a context which influences the acceptability and effectiveness of that intervention that can determine whether a user may or may not want to comply with the recommendations provided by a contact tracing app or even participate in contact tracing altogether (e.g., by not downloading the app).

As of October 2021, less than a third of the UK population were estimated to have downloaded the COVID-19 app and it was acknowledged that some people were choosing to not use it (Committee of Public Accounts, 2021). Findings from 1001 responders to the survey deployed by this research group found over half (*n* = 511) had not downloaded the app, whom also had significantly lower trust than those who had downloaded the app (Dowthwaite et al., 2022). It cannot be assumed that the public will use something because it has been developed with governmental or health services. The data presented here highlight that members of the public want technology to be safe to use i.e., to comply with legislation regarding their personal data yet, previous attempts at digitisation by the NHS are questionable regarding data protection. For example, an assessment of apps that were accredited for safe use for patients and the public were held on the NHS Health Apps Library from 2013 to 2021 (Digital National Health Service, 2022) found two thirds did not have a privacy policy, half the apps were sending personal data without encryption and 10 % of apps sent unencrypted usernames and passwords (Huckvale et al., 2015). Misuse of personal data has been identified as a major concern relating to trust in technology (Toreini et al., 2020), and of greater importance for this setting as privacy has been highlighted as a major concern relating to T&T systems (Williams et al., 2020). To be trusted, governmental bodies such as the NHS must be seen to uphold legislation and public values, however concerns that the government were not responsible around privacy and personal data were reasons for hesitancy in downloading the COVID-19 app (Orzech et al., 2018). The definition of trust in healthcare systems highlights the importance of a wider context, also accounting for trust of the government or state which might have influence on how an individual may trust an app such as the COVID-19 app. This article calls for further research into defining and understanding public trust which will come with guidance on how to better measure and build trust (Taylor et al., 2023).

Digitising medical healthcare necessitates even greater public trust due to the increased risk and uncertainty presented by health problems (Calnan and Rowe, 2006), however the results of this study also recognise the role the media may have had in creating doubt. News outlets and social media were felt to have a role in influencing participants' perceptions of the COVID-19 app, even prior to it being made available to the public. Social media has been held responsible for mass sharing of misinformation during the pandemic, which can result in people not adhering to public health recommendations (Laato et al., 2020). A longitudinal study repeating the survey deployed by this research group explored the perception and use of the app over time and identified the media as being influential in creating mistrust and negative perceptions of the COVID-19 app (Pepper et al., 2022). The mass misinformation from the pandemic has highlighted the challenge for the digital medical landscape, where patients may feel greater trust in news or social media than in public health systems (Pennycook et al., 2020). Increased distrust in the government may have reduced compliance with healthcare recommendations during COVID-19 (Bollyky et al., 2022) and was reported as the largest contributor to diminishing use of the app in a longitudinal study exploring use of the app and trust over time (Pepper et al., 2022). Furthermore, the elderly were the most likely to believe misinformation relating to COVID-19 (Bapaye and Bapaye, 2021), and BAME groups had lower trust in the NHS and the government and were less likely to download the app to support the NHS (Dowthwaite et al., 2021b). As trust was found to be associated with lower use in the app, this highlights that the most vulnerable to COVID-19 were also those most impacted by factors that might decrease their likelihood to use such an app. Given the need for high trust in the implementation of new technologies (Pacifico Silva et al., 2018) and the need for high trust in the compliance during T&T programmes (Siegrist et al., 2021; Siegrist and Zingg, 2014), the media is also likely to have played a role in unsettling the public view of the T&T app. Therefore, consideration should be given not only to the design of new technologies but also in the way they are promoted by media channels given their influence over trust in healthcare.

Despite currently being post-pandemic, themes around the implementation of technology and trustworthiness of the NHS and government remain topical given the increasing digitisation of the NHS. There are major plans for technology advances in the NHS, including 20 % of the workforce being affected by Digital Medicine, Genomics, Artificial Intelligence, and Robotics by 2025 (National Health Service, 2019) and the use of technology as a solution for health services for a range of conditions and for a variety of aims, from admin, prevention to intervention (Murray et al., 2016; British Medical Association, 2019). The NHS is continuing to battle with staff shortages and insufficient funding to improve access to care for those on waitlists (Alderwick, 2022) and technology is being heralded as a potential answer to reduce unnecessary workloads and reduce delays (British Medical Association, 2019). However, recent reports relating to problems implementing the new COVID-19 app (Best, 2019) echoes back to the NHS not being capable of producing safe and reliable digital health care. Responses from the survey associated with these interviews showed higher trust was associated with perceived understanding, usefulness, and ease-of-use of the COVID-19 app (Dowthwaite et al., 2022). Further work on understanding public adoption of the T&T app found a lack of understanding of how the app worked was highest in those who did not trust the app, and BAME communities had the lowest rates of uptake and use (Dowthwaite et al., 2021b). If technology does offer an opportunity for healthcare services to better meet demand, it must be done in a way that instils trust in the public and ensures their cooperation with technological innovation. Those developing digital healthcare products should consider how the public understands a medical device, both in terms of how it works and why it might be useful. These actions could support trust-building in technology that might increase uptake and continued use of such interventions.

Evidence suggests that co-planning and co-design leads to improved compliance of health services, improved service provision and health outcomes (Palumbo, 2016). Guidelines promote the inclusion of the public in the development of technology and research innovations from the very start of the research idea and into the dissemination phases (NIHR, 2019; Craig et al., 2008; Yardley et al., 2015; Mohr et al., 2013). This is especially important when digital technologies are known to cause high levels of disengagement or RRI concerns for the public (Eysenbach, 2005; O'Cathain et al., 2019). Had the public been more involved in giving feedback on how the T&T service was disseminated, the government might have been able to foresee and plan around the difficulties ahead (Department of Health and Social Care, 2020). The current research findings highlighted how the build of the COVID-19 app may have improved engagement with the app, such as a step count, the ability to share information with others or, accessing local resources about COVID-19. They also highlighted generic concerns with using an automated system to track and trace the public, and general uptake of the COVID-19 app suggests that the wider public also had reservations with the system. Considering the vast plans to increase the use of digital technology within the medical sector, it is vital that the public are continuously involved throughout the design and development of an intervention to ensure it meets the users' requirements (Achilles et al., 2020). Approaches that integrate RRI and PPI, can increased trust and confidence in new digital innovations and therefore should be included as standard in the development of new innovations.

## 6 Limitations

The number of participants in the study could be considered on the lower end of the recommendation of 10–20 participants for a medium-sized study (Clarke and Braun, 2013), nonetheless the data was felt to be rich enough by the research team to complete thematic analysis with and was a pragmatic decision to move onto the formation of the questionnaire (Dowthwaite et al., 2021a). Interviews were conducted prior to the deployment of the COVID-19 app and we therefore do not know how these participants feel after having used the app. Nonetheless, these interviews give a snapshot of how people were expecting to feel and provides us with information about how negative feelings toward future automated systems could be overcome. Additionally, participants in these interviews were self-selected and recruited from a UK context so we cannot ascertain how such thoughts and opinions would extend outside of a UK context, particularly given the uniqueness of the NHS and political systems. Furthermore, an error during data collection meant that the demographic variable ‘age’ was not collected which would have been useful for better understanding this sample.

## 7 Conclusions

Overall, participants outlined the need for different user requirements that could be built into the system that would support increased adherence with T&T in the UK. Concurrently, participants raised questions surrounding personal data and privacy of their data, plus the level of automation versus nonautomation, and the impact this could have on how much they trusted the system. Additionally, themes highlighted that the use of tracking technology does not happen within a vacuum, but within a pre-existing environment influenced by variables including media and perception of other's compliance to T&T.

## Declaration of competing interest

The authors declare that they have no known competing financial interests or personal relationships that could have appeared to influence the work reported in this paper.

## Data Availability

Due to the personal nature of this topic, supporting data is available with conditions. Further information about the data and conditions for access are available at the University of Nottingham data repository (doi:10.17639/nott.7401).

## Appendix A Supplementary data

Appendix 1 COREQ checklist. Appendix 1

Appendix 2 Interview schedule. Appendix 2

Appendix 3 Supporting quotes. Appendix 3
