# Supplementary material for: Exploring individual's public trust in the NHS Test and Trace System – A pragmatic reflexive thematic analysis
Source: Internet Interv. 2024 Apr 4;36:100740. doi: 10.1016/j.invent.2024.100740 (PMC11021953; doi:10.1016/j.invent.2024.100740)
Supplement: Appendix 2 — Interview schedule. [file mmc2.docx]

# Appendix 1 - Interview Guide

Key thing to keep in mind: interview purposefully exploratory and open-ended, all answers should be probed for clarification of reasoning – ie ‘why?’-type follow ups.

1. What are your thoughts or feelings about NHS Test and Trace?
   1. Do you have any specific concerns about it?
   2. What is your understanding of how it works?
   3. What role do you think automation plays in this?
   4. What role do you think human decision-making plays in this?
   5. How would you feel if you got a call from a tracer and they told you to self-isolate for 14 days? What would you do/say?
2. What are your thoughts the Test and Trace App?
   1. What is your understanding of how it works?
   2. Are you aware of any debates in the media about the app?
   3. Do you have any view on the involvement of big companies in the making of the app?
   4. Would you use the app if it was available [or if it is:] Do you already use the app or are you intending on downloading it? If not, why? [aiming at finding out barriers to use]
   5. How would you feel if the app notified you told you to self-isolate for 14 days? What would you do?
3. Have you heard anything about contact tracing in other countries?
4. What are your thoughts on the Government’s handling of the outbreak?
5. What about the NHS, and its role in handling the outbreak?
6. Is there anything else we haven’t touched on that you would like to say?
