# Supplementary material for: Exploring individual's public trust in the NHS Test and Trace System – A pragmatic reflexive thematic analysis
Source: Internet Interv. 2024 Apr 4;36:100740. doi: 10.1016/j.invent.2024.100740 (PMC11021953; doi:10.1016/j.invent.2024.100740)
Supplement: Appendix 3 — Supporting quotes. [file mmc3.docx]

## Appendix 1 – Supporting Quotes

## Theme 1: Participants had varied ideas about the system requirements and functionality of T&T

### Subtheme 1a: T&T needs to feature validation or verification processes to confirm both the user and the system are authenticated

*“I think if I was happy with where the app was from as then I would use, I would use that” (J55)*

“*I think you should get an alarm, you should log in, confirm who you are, who you say you are using the phone and then the details come up” (H57)*

### Subtheme 1b: The app should include other sources of information and integration

“*step count*” *(G129)*

*“you would hope that there would be something in the app that would send you in the right direction, like: do this, talk to this person, order this”*

### Subtheme 1c: Verification to check the accuracy of exposure for both phone and app tracking were wanted

“*I'd like it to be: at this time you came into contact with somebody at this place; self-isolate”* (K103) *you were in the Lions head, wherever, on this day, then I probably [would] have more confidence in it”* (K33)

“*is it gonna [sic] go on a small distance? Or how long?”* (I57)

“*the more you can assume error then more you'd be likely to just ignore it*” (G97)

## Theme 2: There are concerns that people will not comply with T&T, explained by various personal and structural elements

“*you can't necessarily trust that people will do that*” (I3)

### Subtheme 2a: Despite the impact on life, most people who were asked to track and isolate would do so to protect others

“greater good” (C12)

“*if I have for example, popped to see my mom and dad, as careful as we are being, in between that person, you know, realising they may have symptoms getting the test and a positive result, and then someone contacting me*” (C33)

“*I would definitely want to self-isolate”* (L35).

### Subtheme 2b: Face-to-face informing of persons to isolate was felt to go against social distancing guidelines, although would increase compliance

“*I'd be more concerned about people moving around transmitting more than anything, so I’d be much more comfortable with a phone call to be honest*” (F17).

## Theme 3: Participants had varied concerns about the use of personal data in track and tracing systems, yet the pandemic made many people concede their concerns

### Subtheme 3a: Data concerns about T&T revolved around how data would be used now and, in the future, who by and potential hacks

“*my trust issue is around where that data is gone and who's collected it and for what purposes and how I opt out and how I get, and that my trust, my absolute trust concerns with it in terms of what it's trying to do”* (A65)

“*I think it has to be very clear that this is only used, you know there has to be certain regulations which that this is only used for this purpose, and… once this pandemic is potentially over, that has to stop”* (I83).

### Subtheme 3b: Data concerns could be reduced by limiting the personal data taken and be improved with rules

“*maybe if there is a way of just finding out the places you visited rather than you, the places, your movements you know*” (L47)

*“I would be put off if I were left to do [identity verification using a piece of photographic ID] on test and trace app, yeah?”* (J71)

*“I would want reassurance from independent people that, uh, it is totally secure and that Google and Apple or anybody else of that ilk who are involved do not get to collect my personal data*” (B47)

### Subtheme 3c: Many people are resigned to their data already being held by technology companies so the pandemic did not increase their concerns

*“I presume Big Brother is watching us all the time and there is some way of finding out*” (D5)

“*I think that's [not wanting to be tracked and violation of human rights] quite naive 'cause it's happening through the mobile phones all the time anyway”* (E37)

“*I see it [as] quite low risk*” (C7)

“*I'm well aware that my data is being harvested constantly what I do… so I'm quite aware that data from me will be used, um I don't have a strong feeling*” (C71).

## Theme 4: Trust in human and automated approaches to test and trace differs

“*T&T relies on trust*” (G47).

### Subtheme 4a: Automation in the system increases capacity and reduces human errors

“*I think, oddly enough, I might trust it more than, uh, than an individual ringing me up or coming to my door*” (B63)

“*having as much time as possible to take action for my friends and family and others around is more important to me than a human ringing me*” (C103)

“*I wouldn't say I fully trust [a manual approach] purely because of the human error side of things*” (F19)

“*I think the hardest part is the inconsistency of different places have got different things, so yes, if there was an app and it kind of tracks and chase you automatically, then that seems quite sensible*” (D69)

“*if it was an app that you could press the app to locate the people or they have been in contact or something. I think there's more chance to make possibly or people do that 'cause it's easy*” (I53).

### Subtheme 4b: A human approach provides compassion and tailored responses

“*trust it, if it was entirely automated? No no*” (L87)

*“it just seems like the worst way to deliver a kind of significant news. You know, it's kind of almost like unemployment by text message*” (A27)

“*I completely get it's a massive task and they will need some level of automation, um, but if someone is going to ring you to say no need to worry just… It would be nice*” (C43)

“*if you had an app then you could have a follow up phone call if you could, if you requested it on the app so that you can get a bit of reassurance and know exactly what's going on from a human being*” (I61).

## Theme 5: The media coverage added to people's negative perception about T&T

Themes show the role of the media in how opinions were developed and formed related to T&T. This was especially true for becoming more aware of the problems surrounding T&T (subtheme 5a), a lack of up-to-date information (subtheme 5b) and social media as a source of unreliable information (subtheme 5c). A negative perception was also felt to be compounded by comparisons with different countries, who appeared to be handling covid better than the UK.

### Subtheme 5a: The media highlighted the problems and lack of effectiveness of the T&T system

*“[the] little bits that I've read in the media about it, about the engagement and the amount of success it’s having is a little bit worrying”* (J3)

“*whether our method is going to beat other countries and or whether it's of its previous standard”* (K65)

“*generally people saying that it's not gonna work, it's it's too voluntary, basically”* (H67)

“*from the press and various reports I've read about it, but I get the impression it's not really being particularly applied*“ (A3)

### Subtheme 5b: A lack of coverage and up to date information fostered the uncertainty of the situation

“*I'm not seeing much [on] the news about how effective it's been either, so, not at the moment unfortunately”* (F19).

“*I mostly watch BBC… And yeah, I haven't, haven't heard discussion regarding the app through that*” (E43).

“*know what to expect”* (G43).

“*I'd like to see more adverts to telling people how they actually should wear their masks*” (H199)

“*put more trust in the system from the public as well, possibly and make them using it correctly*” (E91).

### Subtheme 5c: Media, especially social media, features problematic, biased and fake content

*“it's been the typical media thing of cases have doubled, but actually when you look at the underlying numbers the doubling isn't”* (J77)

*“it's just the lack of credible data sources… our TV's biased, or all newspapers have some kind of political sway, so they're going to frame things in a certain way”* (G153)

“*there's so much of it [false information] around at the moment”* (G41)

“*you've got the extreme, and people just don't believe in COVID-19 exists. You’ve got the anti-mask movement already, so I'm I'm following it [the debate] a bit*” (K65).

### Subtheme 5d: The positive handling of COVID-19 abroad made domestic problems more apparent

*“You are safer in Spain at the minute then you are here… I don't think they've [the British government] handled this at all well... look at New Zealand* (H179-180)

“*they must have quite rigorous test and trace system set up there [Australia]”* (E99)

“t*he technological approaches that those two cultures took is they went with a kind of a centralized data collection system in South Korea where that's kind of people aren’t I guess so fearful, not fearful, untrusting of the government”* (A47)

“*something as, unthinking and stupid as a virus, has a dance with the kind of political systems that are in play and the cultural behaviours there”* (A69).
